# Glucosamine inhibits epidermal growth factor-induced proliferation and cell-cycle progression in retinal pigment epithelial cells

**Published:** 2010-12-03

**Authors:** Chang-Min Liang, Ming-Cheng Tai, Yun-Hsiang Chang, Yi-Hao Chen, Ching-Long Chen, Ming-Wei Chien, Jiann-Torng Chen

**Affiliations:** 1 Graduate Institute of Medical Science, National Defense Medical Center, Taipei, Taiwan, Republic of China; 2 Department of Ophthalmology, Tri-Service General Hospital, Taipei, Taiwan, Republic of China; 3 Department of Ophthalmology, School of Medicine, National Defense Medical Center, Taipei, Taiwan, Republic of China

## Abstract

**Purpose:**

To investigate the effects and mechanisms of glucosamine (GlcN) on the proliferation of retinal pigment epithelial cells in response to epidermal growth factor (EGF).

**Methods:**

Cell proliferation was measured in the human retinal pigment epithelial cell line (ARPE-19) cells with the 4-[3-(4iodophenyl)-2-(4-nitrophenyl)-2H-5-tetrazolio]-1,3-benzene disulfonate (WST-1) assay and cell counting. The results were confirmed in human donor cells with the carboxyfluorescein diacetate succinimidyl ester cell proliferation assay (CFSE) cell proliferation assay. In ARPE-19 cells, cell*-*cycle progression was determined by flow cytometry; the protein levels of cell cycle regulators and heat shock protein 90 (Hsp90) were measured by western blotting; the levels and branching of N-glycans were assessed using the L-Phaseolus vulgaris agglutinin lectin-binding assay; and the modulation of N-glycans on EGF receptor (EGFR) was examined by western blotting.

**Results:**

GlcN inhibited retinal pigment epithelium (RPE) proliferation in a dose-dependent manner. During cell-cycle progression induced by EGF, GlcN caused delays at the G_1_–S and G_2_–M transitions without affecting cell viability. GlcN modulated the level and branching of N-glycans on EGFR, suppressed phosphorylation of EGFR, and reduced phosphorylation of extracellular signal-regulated kinases, erine/threonine protein kinase, and the signal transducer and activator of transcription 3 (STAT3). GlcN had only minor effects on the expression of Hsp90, Grp78, and transcription factor CHOP/GADD 153 markers of nonspecific stress in the endoplasmic reticulum.

**Conclusions:**

GlcN effectively suppressed proliferation of RPE cells in vitro. This effect appeared to be achieved through modification of N-glycans on EGFR. Further research into the role of GlcN as a potential agent for the prevention and treatment of RPE-mediated ocular proliferative disorders, such as proliferative vitreoretinopathy, and other EGF-dependent proliferative cell-growth disorders, is warranted.

## Introduction

Proliferative vitreoretinopathy (PVR) is the most common cause of treatment failure in rhegmatogenous retinal detachment [1]. The mechanisms underlying the pathogenesis of PVR are unknown, but are presumed to include either sustained or discordant growth-factor responses that accelerate the proliferation, migration, and contraction of the retinal pigment epithelium (RPE) [2]. Accumulating evidence indicates that epidermal growth factor (EGF)–EGF receptor (EGFR) signaling is involved in diverse cellular processes, including the growth, differentiation, and survival of RPE cells in vitro [3–9]. Furthermore, the activation of EGF–EGFR signaling seems to be an important feature of the pathogenesis of PVR [10–12].

Our previous studies have shown that glucosamine (GlcN) has an anti-inflammatory effect in ocular inflammatory disorders [13,14]. In addition, GlcN has been reported to inhibit the growth of various cell types [15]. Because GlcN is an inhibitor of the biosynthesis and processing of N-linked oligosaccharides and causes dramatic and reversible changes in the nature of the lipid-linked oligosaccharides of glycoproteins [16], we hypothesized that GlcN might exert an antiproliferative effect on human retinal pigment epithelial cell line (ARPE)-19 cells and that reduced branching and levels of N-glycans on surface growth-factor receptors might be involved in the mechanism. Demonstrating the validity of this hypothesis could provide support for the use of GlcN as a potential agent for the prevention and treatment of RPE-mediated ocular proliferative disorders, such as PVR. The purpose of the study, therefore, was to examine the effects and mechanism of action of GlcN on EGF-induced proliferation, in vitro, in human donor cells and ARPE-19 cells, respectively.

## Methods

### Cell culture

ARPE-19 cells were obtained from the American Type Culture Collection (Manassas, VA) and maintained in Dulbecco’s modified Eagle’s medium (F-12) supplemented with 4 mM L-glutamine, 10% fetal bovine serum (FBS), 100 U/ml penicillin, and 100 mg/ml streptomycin at 37 °C in 5% CO_2_ in air. The culture medium was replaced twice weekly.

### Cytotoxicity assay

ARPE-19 cells were seeded into 24-well plates at a density of 2×10^4^ cells per well in 1 ml Dulbecco’s modified Eagle’s medium and 10% FBS. The medium was changed after 24 h, and GlcN was added in concentrations between 0 mM and 140 mM. After 24 h, ARPE-19 cells were trypsinized and stained with 2% trypan blue (1:1 vol/vol) for 5 min. Viable (unstained) and dead (stained) cells were counted from each well by hemocytometer. Experiments were performed in triplicate and repeated three times. At least 400 cells were counted in each well.

### Proliferation assays

#### 4-[3-(4iodophenyl)-2-(4-nitrophenyl)-2H-5-tetrazolio]-1,3-benzene disulfonate cell proliferation assay

The cell proliferation test was based on the ready-to-use cell proliferation reagent 4-[3-(4iodophenyl)-2-(4-nitrophenyl)-2H-5-tetrazolio]-1,3-benzene disulfonate (WST-1; Roche Diagnostics, Indianapolis, IN). After treatment for 48 h with various concentrations of GlcN in serum-free medium with 10 ng/ml EGF stimulation, 10 μl of WST-1 reagent were added to the medium in each well. The cells were incubated in a humidified atmosphere at 37 °C in 5% CO_2_/95% air for 1 h, the multititer plate was shaken thoroughly for 1 min, and absorbances were read at 450 nm. The background absorbance was measured in wells containing only the dye solution and culture medium. Cell proliferation data were obtained from at least three experiments with at least six wells at each concentration in separate 96-well plates. The mean optical density values corresponding to the untreated controls were taken as 100%. The results were expressed as the percentage of the optical density of treated cells relative to that of untreated controls.

#### Cell counting

During stimulation with 10% FBS or 10 ng/ml EGF, ARPE-19 cells were treated with 2.5 mM or 5 mM GlcN for 1–5 days. At the end of the treatment period, the cells were trypsinized and washed twice with ice-cold phosphate buffer solution (PBS; 137 mM NaCl, 2.7 mM KCl, 100 mM Na_2_HPO_4_, 2 mM KH_2_PO_4_, pH 7.4). For each sample, an aliquot of cells was counted using a hemocytometer to determine the cell number.

#### Carboxyfluorescein diacetate succinimidyl ester cell proliferation assay

For staining with cell proliferation assay (CFSE; Invitrogen*/*Molecular Probes, Eugene, OR), 1×10^7^/ml human donor RPE cells in PBS were incubated at 37 °C for 15 min with 1.0 μM CFSE in 0.1% FBS/PBS. The human donor RPE cells were obtained from the cryopreserved cells used in our previous study [11]. Staining was terminated by the addition of culture medium containing 10% FBS. The cells were washed once in 10% FBS/PBS and resuspended in culture medium at 2×10^6^/ml. Stained cells (2×10^5^/well, 100 μl) were cultured overnight in 60 mm dishes with culture medium containing 20% FBS. The medium was then exchanged with one containing 10 ng/ml EGF with 2.5 mM, 5.0 mM GlcN, or 30 mM glucose, and incubated for 3 days. At the end of the treatment period, the cells were trypsinized and analyzed on a FACScan flow cytometer (Becton, Dickinson, & Co., Sunnyvale, CA).

The CFSE passively diffuses into cells. It is colorless and nonfluorescent until the acetate groups are cleaved by intracellular esterases to yield highly fluorescent carboxyfluorescein succinimidyl ester. The succinimidyl ester group reacts with intracellular amines, forming fluorescent conjugates that are well retained and can be fixed with aldehyde fixatives. The dye–protein adducts that form in labeled cells are retained by the cells. The label is inherited and shared by daughter cells after cell division, and is not transferred to adjacent cells in a population. Therefore, the cells undergoing the processes of cell division show gradually decreasing intensity of fluorescence, and the lower level of staining indicates a rapidly proliferating cell population.

### Cell viability assays

#### Detection of apoptosis

Apoptosis-mediated death of GlcN-treated cells was examined by a double-staining method with fluorescein isothiocyanate (FITC)-labeled annexin V (Invitrogen, Carlsbad, CA)/propidium iodide (PI; Sigma-Aldrich; St. Louis, MO). ARPE-19 cells were starved by culture in serum-free medium for 24 h and then were stimulated with EGF (with or without treatment with GlcN for 5 days). We changed the medium each day. After treatment with 2.5 mM or 5.0 mM GlcN, the cells were trypsinized and counted. A fraction of the cells (2×10^5^) was collected by centrifugation, and the pellet was washed twice with PBS. The cell pellet was resuspended, incubated for 15 min in 100 μl of labeling solution (5 μl of annexin V in 100 μl of Hepes buffer [10 mM Hepes/NaOH pH 7.4, 140 mM NaCl, 5 mM CaCl_2_]), then collected by centrifugation and washed twice with PBS. Finally, 400 μl of Hepes buffer containing 2.5 μl of PI (50 μg/ml) was added, and the samples were analyzed on a FACScan flow cytometer.

### Cell-cycle analysis

ARPE-19 cells were treated with GlcN for 24 h in serum-free medium, and the cell cycle was arrested by incubation for 24 h in culture medium containing aphidicolin (1 μg/ml; Sigma-Aldrich). The cells were then released from arrest by incubation in drug-free culture medium containing 10 ng/ml EGF and harvested with trypsin at various time points, washed twice with PBS, fixed in ice-cold 70% (v/v) ethanol, and stored at 4 °C until use. Before flow-cytometric analysis, the cells were washed with PBS and centrifuged, and the cell pellets were resuspended in RNase (1 mg/ml) for 30 min. The cells were then stained for 15 min with PI in PBS (final concentration 40 μg/ml) before analysis with a FACScan flow cytometer using CellQuest software (Becton, Dickinson, & Co.)

### Western blot analysis

At the end of the treatment period, the cells were washed twice with PBS and detached by scraping. The cells were pelleted at 1000× g, resuspended, and sonicated in cold lysis buffer (50 mM Tris-HCl [pH 7.5], 2% sodium dodecyl sulfate [SDS], 1 mM phenylmethylsulfonyl fluoride, and 10 μl/ml protease inhibitors). The lysates were centrifuged at 12,000× g for 10 min, and the clear supernatant was removed into fresh Eppendorf tubes. The total protein was estimated using the Pierce bicinchoninic acid (BCA; Pierce, Rockford, IL) protein assay. The samples (20 μg of lysate) were then boiled for 5 min, loaded onto a sodium dodecyl sulfate/10% polyacrylamide gel, separated electrophoretically, and transferred to a polyvinylidene difluoride (PVDF) membrane (Immobilon; Millipore Corp., Bedford, MA). The membranes were blocked with 5% (w/v) milk in Tris-buffered saline (50 mM Tris.HCl, pH 7.4 and 150 mM NaCl) containing 0.05% Tween-20 (TBST) for 60–120 min at room temperature on a shaking table. The blots were incubated for 60 min at room temperature with primary antibody against the following: cyclin A, cyclin D1, cyclin E, retinoblastoma protein (Rb), p-RB (Ser 807/811), AKT, phosphorylated AKT (Ser473), STAT3, phosphorylated STAT3, and EGFR (all antibodies from BD PharMingen, San Diego, CA); CDK2 and CDK4 (diluted 1:1,000 in TBST; Santa Cruz Biotechnology, Santa Cruz, CA); ERK1, ERK2, and phosphorylated ERK1/2 (diluted 1:2,000 in TBST; BD PharMingen); Hsp27, Hsp70, and Hsp90 (diluted 1:1,000 in TBST; Santa Cruz Biotechnology); Grp78 and CHOP-GADD (diluted 1:1,000 in TBST; Santa Cruz Biotechnology); total EGFR and active EGFR (diluted 1:1,000 in TBST; BD PharMingen); β-catenin (diluted 1:2,000 in TBST; Santa Cruz Biotechnology); or glyceraldehyde-3-phosphate dehydrogenase (diluted 1:20,000 in TBST; Rockland Immunochemicals, Gilbertsville, PA). After extensive washing, the membranes were blotted with horseradish-peroxidase-conjugated secondary antibody (1:1,000; Jackson ImmunoResearch Laboratories, West Grove, PA) for 1 h at room temperature. The peroxidase activity on the membrane was visualized on X-ray film by a standard enhanced chemiluminescence procedure.

### Flow-cytometric analysis of EGFR activation

Phosphorylated EGFR (p-EGFR) and total EGFR levels were quantitatively measured by flow cytometry with BD Phosflow reagents (BD Biosciences) according to the manufacturer’s protocol. Briefly, 1×10^6^ cells were cultured in serum-free medium containing 30 mM glucose, 2.5 mM GlcN, or 5.0 mM GlcN for 24 h. The cells were then stimulated with 10 ng/ml EGF at 37 °C for 30 min and harvested with PBS-based enzyme-free dissociation buffer (Invitrogen). After centrifugation, the cell pellets were washed twice in PBS and immediately fixed for 10 min at 37 °C by the addition of an equal volume of Phosflow Fix Buffer I. After centrifugation, the cells were permeabilized with 1 ml of BD Phosflow Perm Buffer III, incubated for 30 min on ice, and washed twice with stain buffer. The cell pellet was resuspended in 100 μl of stain buffer containing the primary antibody. The following primary antibodies were used: phycoerythrin (PE)-conjugated anti-total-EGFR (cells were not permeabilized for total EGFR detection) and Alexa-Fluor-647-conjugated anti-p-EGFR (Y845; BD PharMingen). After incubation for 30 min in the dark, the cells were washed twice with 1 mL of stain buffer before analysis performed by FACScan flow cytometer.

### Western blot analysis of EGFR activation

ARPE-19 cells were cultured to 80% confluence. The medium was then changed, and the cells were cultured with the addition of 2 μg/ml tunicamycin, 30 mM glucose, or 5 mM GlcN in serum-free medium for a further 24 h. Then the cells were stimulated with EGF for 5 min. At the final time point, proteins contained in the whole cell lysates were analyzed by western blotting.

### Western blot analysis of β-catenin expression

ARPE-19 cells were cultured to 80% confluence. The medium was then changed, and the cells were cultured with the addition of 2.5 mM or 5 mM GlcN in serum-free medium for a further 24 h. Then the cells were stimulated with EGF for 24 h. At the final time point, proteins contained in the whole cell lysates were analyzed by western blotting with probes against β-catenin.

### Lectin-binding analysis

Phytohemagglutinin-L (L-PHA) is a plant lectin that specifically binds β-1,6-GlcNAc-branched N-glycans. Flow-cytometric measurement of L-PHA binding to ARPE-19 cells was used to characterize the branching of N-linked oligosaccharides on surface proteins. Cells with increased metabolic flux through the hexosamine pathway (i.e., treated with 30 mM N-acetyl-glucosamine [GlcNAc] or 30 mM glucose) were used as a positive control. Cells treated with tunicamycin, an inhibitor of protein N-glycosylation, were used as the negative control. ARPE-19 cells were cultured to 80% confluence. The medium was then changed, and the cells were cultured with the addition of 2 μg/ml tunicamycin, 30 mM GlcNAc, 30 mM glucose, 2.5 mM GlcN, or 5 mM GlcN in medium containing 10% FBS for a further 24 h. The cells were then harvested with PBS-based enzyme-free dissociation buffer (Invitrogen). After centrifugation, the cell pellets were rinsed, resuspended in PBS, and incubated with FITC–L-PHA (10 μg/ml; Vector, Burlingame, CA) and 1% BSA (BSA) in PBS on ice for 15 min. After the cells had been washed once with four volumes of 1% BSA/PBS, they were analyzed on a FACScan flow cytometer.

### Markers of stress in the endoplasmic reticulum

ARPE-19 cells were treated with 30 mM glucose, 5 mM GlcN, or 1 μM thapsigargin as the positive control. The levels of expression of Hsp90, Grp78, and CHOP-GADD were determined by western blotting after treatment for 24 h and 48 h.

### Statistical analysis

All data are expressed as mean±standard deviation (SD). The difference in inhibition of ARPE-19 cell proliferation after treatment with the various concentrations of GlcN was compared with one-way ANOVA (ANOVA). The ARPE-19 cell count observed at each day was also examined with one-way ANOVA. When significant results were revealed by one-way ANOVA, post-hoc analyses were performed with Tukey’s test. Linear regression analysis was performed to test the time-trend of the cell count. In addition, two-way ANOVA was performed to check whether the slopes of the time-trend curves were different among the control cells and among those treated with 2.5 mM and 5.0 mM GlcN. Two-sided comparisons were implemented with SPSS 15.0 software (SPSS Inc., Chicago, IL) and evaluated at the 0.05 level of significance.

## Results

### Cytotoxicity of glucosamine

**Figure 1:**
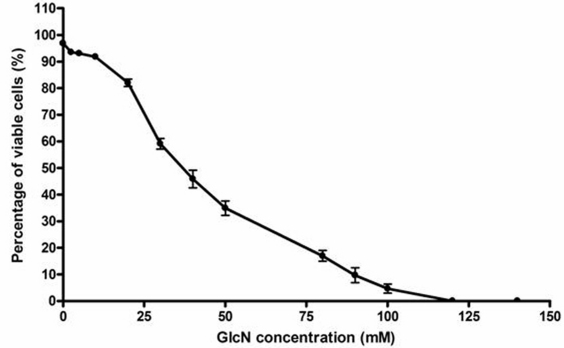
Viability of human retinal pigment epithelial cell line (ARPE-19) cells and determination of the LC_50_ concentration in the presence of GlcN. ARPE-19 cells were cultured in Dulbecco’s modified Eagle’s medium and 10% fetal bovine serum (FBS). The medium was changed after 24 h, and glucosamine (GlcN) was added in concentrations between 0 mM and 140 mM. After 24 h, viability was determined with 2% trypan blue. The LC_50_ was 39.0 mM. An LC_50_ value is the concentration of a material that will kill 50% of the test cells.

The results of the trypan blue exclusion assay to determine the lethal concentration (LC)_50_ of GlcN are shown in Figure 1. The LC_50_ concentration was 39.0 mM, and approximately 3% and 6.5% cytotoxicity was observed with concentrations of 2.5 mM and 5 mM, respectively.

### Suppression of epidermal growth factor-induced proliferation of RPE cells by GlcN in vitro

**Figure 2:**
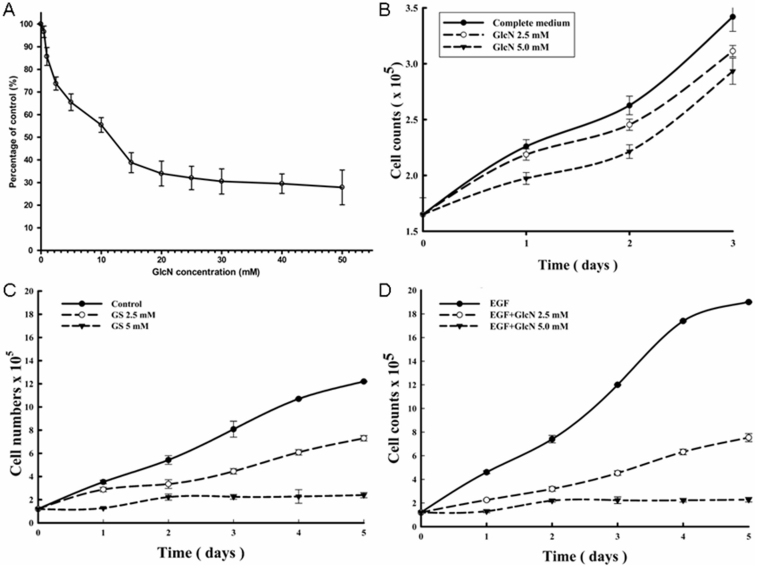
Inhibition of human retinal pigment epithelial cell line (ARPE-19) cell proliferation by glucosamine (GlcN), measured with a colorimetric test (WST-1) and cell counting. **A**: ARPE-19 cells were cultured for 48 h at different concentration of GlcN. The proliferation results are expressed as a mean percentage of control proliferation. **B**: After 24 h culture, the ARPE-19 cells were exposed to 2.5 mM or 5.0 mM GlcN for 3 days. During this time, the medium was not changed. The ARPE-19 cells were exposed to 2.5 mM or 5.0 mM GlcN with either 10% fetal bovine serum (FBS; **C**) or 10 ng/ml endothelial growth factor (EGF; **D**) stimulation for 5 days (the medium with fresh GlcN included was changed daily). The results are the means±SD of five independent experiments. The difference in inhibition of ARPE-19 cell proliferation among various concentrations of GlcN was compared with one-way ANOVA (ANOVA); Tukey’s test was used for post-hoc analyses. * p<0.05, ** p<0.01, and *** p<0.001 versus the control.

As shown in Figure 2A, cell proliferation was significantly lower in cells treated with GlcN at concentrations of 1.0 mM or higher (all p<0.05) than in control cells. The proliferation was reduced by 3.4%, 14.3%, 26.3%, 34.5%, and 44.6% at 0.5 mM, 1.0 mM, 2.5 mM, 5.0 mM, and 10.0 mM GlcN, respectively. The IC_50_ concentration was 13.6 mM (Figure 2A). All further experiments were conducted with 2.5 mM and 5.0 mM GlcN because these concentrations had a relatively large effect on proliferation but were minimally cytotoxic.

To further explore the effects of GlcN on proliferation of RPE cells, ARPE-19 cells were cultured in medium containing 0, 2.5, or 5.0 mM GlcN for 3 days. The slope of the growth curve decreased significantly during the first day after treatment, but gradually returned to parallel the slope of the control cells (Figure 2B). The uptake of GlcN by the cells led to a reduced concentration of GlcN in the medium and a tapering of the antiproliferative effect. Therefore, we changed the medium (including fresh GlcN) each day and stimulated proliferation with 10% FBS (Figure 2C) or EGF (10 ng/ml, Figure 2D). Except for the measurement at day 2, significant differences in the number of cells among the three groups were found (all p<0.05) after stimulation with EGF (Figure 2D). By day 5 in the presence of EGF, the cell counts were reduced by 60.5% and 88% with 2.5 mM and 5.0 mM GlcN, respectively.

### The antiproliferative effect of glucosamine is not related to apoptosis

**Figure 3:**
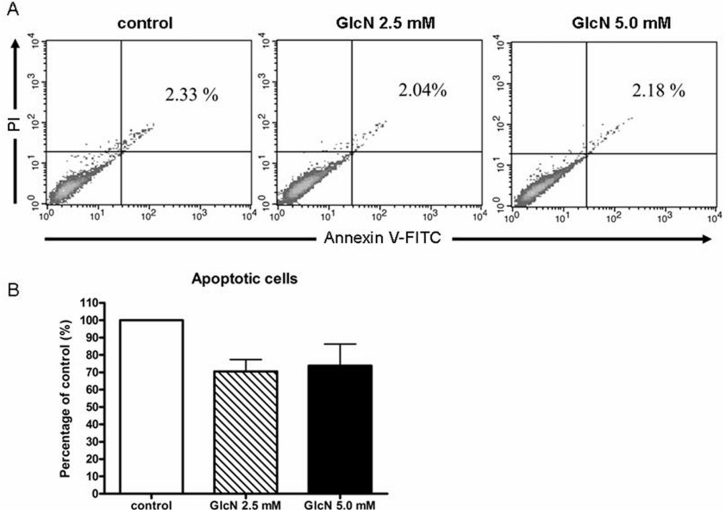
Effects of glucosamine (GlcN) on human retinal pigment epithelial cell line (ARPE-19) cell viability shown by calcein/PI flow cytometric analysis. **A**: ARPE-19 cells were treated for 5 days with 2.5 mM or 5.0 mM GlcN under serum-free conditions, and apoptotic cells were measured as the percentage of annexin V-positive*/*PI*-*positive cells. **B**: The quantitative data collected from the fluorescent images are expressed as the mean percentage±SD from three individual experiments.

To investigate whether the antiproliferative effect of GlcN was related to apoptosis, we used annexin V/PI staining (Figure 3A,B) to measure apoptosis after 24 h of treatment with 2.5 mM or 5.0 mM GlcN under serum-free conditions. The results showed that GlcN did not induce apoptosis in the ARPE-19 cells.

### GlcN delays EGF-induced cell-cycle progression

**Figure 4:**
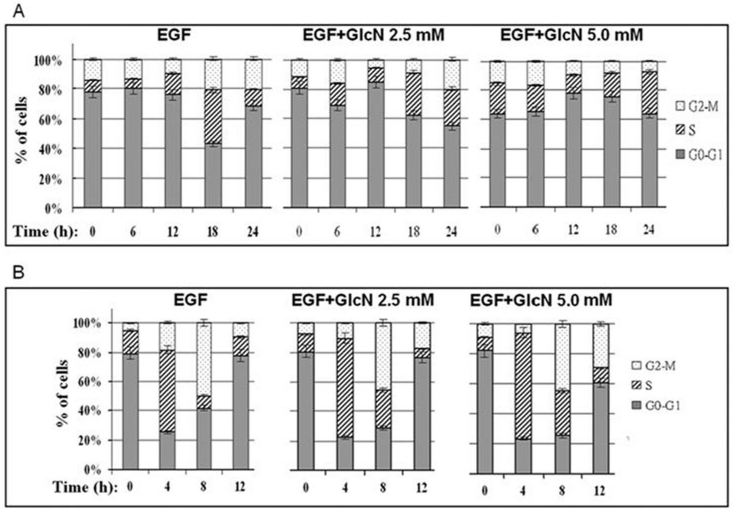
Effects of glucosamine (GlcN) on epidermal growth factor (EGF)-induced cell-cycle progression in human retinal pigment epithelial cell line (ARPE-19) cells. **A**: ARPE-19 cells were deprived of serum for 24 h and then harvested at the indicated times after stimulation with 10 ng/ml EGF with or without the addition of GlcN. B: ARPE-19 cells were synchronized in early S phase by serum deprivation and aphidicolin treatment and harvested at the indicated times after release from the aphidicolin block by stimulation with 10 ng/ml EGF with or without the addition of GlcN. The percentages of cells in the G_0_–G_1_, S, and G_2_–M phases were determined. The data are the means±SD of three independent experiments.

We next examined the effects of GlcN on the progression of RPE cells through the cell cycle after the cells had been synchronized in G_0_–G_1_ phase. At 18 h after stimulation with EGF, ~36% of the ARPE-19 control cells had entered S phase, whereas ~29% of the cells treated with 2.5 mM GlcN and only ~17% of the cells treated with 5.0 mM GlcN had done so (Figure 4A and Table 1). At 24 h, ~20% of the control cells and the cells treated with 2.5 mM GlcN progressed to G_2_–M phase, although only ~25% of the cells treated with 2.5 mM GlcN were still in S phase (Figure 4A). In contrast, ~28% of the cells treated with 5.0 mM GlcN remained in S phase at 24 h, and very few (<10%) had entered G_2_–M phase. A similar slowing of cell-cycle progression was observed when the cells were synchronized in S phase rather than G_0_–G_1_ phase (Figure 4B and Table 2).

**Table 1:** Percentage of human retinal pigment epithelial cell line (ARPE-19) cells in each cell-cycle without aphidicolin treatment (cells synchronized in G_0_–G_1_)

**Cell cycle phase**	**EGF Mean±SD**	**EGF+GlcN 2.5 mM Mean±SD**	**EGF+GlcN 5.0 mM Mean±SD**
**G_0_/G_1_**			
0 h	77.57±3.88	81.06±4.05	63.67±3.18
6 h	80.33±4.02	69.13±3.46	65.51±3.28
12 h	75.87±3.79	84.74±4.24	77.89±3.89
18 h	42.86±2.14	62.58±3.13	75.65±3.78
24 h	68.66±3.43	55.18±2.76	64.34±3.22
**S**			
0 h	8.24±0.41	7.2±0.36	21.18±1.06
6 h	6.44±0.32	14.48±0.72	18.22±0.91
12 h	14.52±0.73	9.65±0.48	12.68±0.63
18 h	36.21±1.81	28.75±1.44	16.61±0.83
24 h	11.0±0.55	24.89±1.24	28.46±1.42
**G_2_/M**			
0 h	14.18±0.71	11.74±0.59	15.14±0.76
6 h	13.23±0.66	16.39±0.82	16.27±0.81
12 h	9.61±0.48	5.61±0.28	9.43±0.47
18 h	20.94±1.05	8.68±0.43	7.75±0.39
24 h	20.34±1.02	19.93±1	7.19±0.36

Abbreviations: EGF represents epidermal growth factor; SD represents standard deviation.

**Table 2:** Percentage of human retinal pigment epithelial cell line (ARPE-19) cells in each cell-cycle after release with aphidicolin treatment

**Cell cycle phase**	**EGF Mean±SD**	**EGF+GlcN 2.5 mM Mean±SD**	**EGF+GlcN 5.0 mM Mean±SD**
**G_0_/G_1_**			
0 h	79.2±3.96	80.66±4.03	82.01±4.1
4 h	25.5±1.27	22.43±1.12	23.27±1.16
8 h	42.09±2.1	29.05±1.45	25.29±1.26
12 h	77.72±3.89	76.63±3.83	60.99±3.05
**S**			
0 h	15.54±0.78	11.66±0.58	9.13±0.46
4 h	55.95±2.8	67.42±3.37	70.79±3.54
8 h	8.03±0.4	25.57±1.28	30.46±1.52
12 h	12.69±0.63	5.95±0.3	9.41±0.47
**G_2_/M**			
0 h	5.27±0.26	7.69±0.38	8.87±0.44
4 h	18.55±0.93	10.15±0.51	5.94±0.3
8 h	49.88±2.49	45.38±2.27	44.25±2.21
12 h	9.59±0.48	17.42±0.87	29.6±1.48

Abbreviations: EGF represents epidermal growth factor; SD represents standard deviation.

### GlcN altered the expression of cyclins and p27

**Figure 5:**
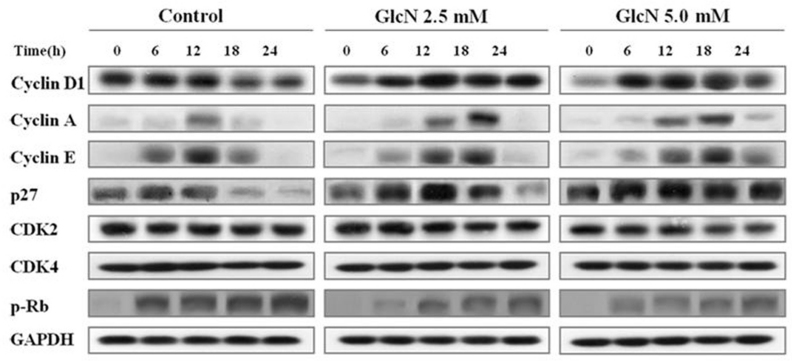
Effects of glucosamine (GlcN) on the expression of cell-cycle regulators. Human retinal pigment epithelial cell line (ARPE-19) cells were deprived of serum for 24 h, stimulated with 10 ng/ml epidermal growth factor (EGF) with or without the addition of GlcN for the indicated times, lysed, and subjected to immunoblotting analysis with antibodies directed against the indicated proteins. The data are representative of at least three independent experiments.

The effects of GlcN on expression of several cyclins, cyclin-dependent kinases (CDKs), and CDK inhibitors were detected; the results are shown in Figure 5. After stimulation with EGF, a marked immediate increase in the amount of cyclin D1 in the control cells was noted, whereas expression of cyclin D1 was delayed in the cells treated with GlcN. Treatment with GlcN also caused the levels of cyclin E, which increase as cells enter G_1_ phase and decline throughout M phase, to decline at a slower rate than in control cells after stimulation with EGF. Stimulation with EGF induced expression of cyclin A, and this was delayed in GlcN-treated cells in parallel with their delayed entry into S phase. Degradation of the CDK inhibitor p27 is required for cell progression through G_0_–G_1_ phase to S phase. However, the abundance of this protein remained higher in GlcN-treated cells than in control cells during their progression through S and G_2_–M phases. The levels and time courses of CDK2 and CDK4 expression were not affected by treatment with GlcN. During the G_1_–S transition, the cyclin D1–CDK4 complex and the cyclin E–CDK2 complex mediate Rb hyperphosphorylation, which results in E2F release and the transcription of growth-associated genes. The 807/811 phosphorylation sites of EGFR were examined because they occur in the region of E2F/pRb binding and may play a key role in the interaction between these two molecules. A reduced level of hyperphosphorylated Rb was observed in the GlcN-treated cells.

### GlcN inhibited phosphorylation of ERK1/2, AKT, and STAT3

**Figure 6:**
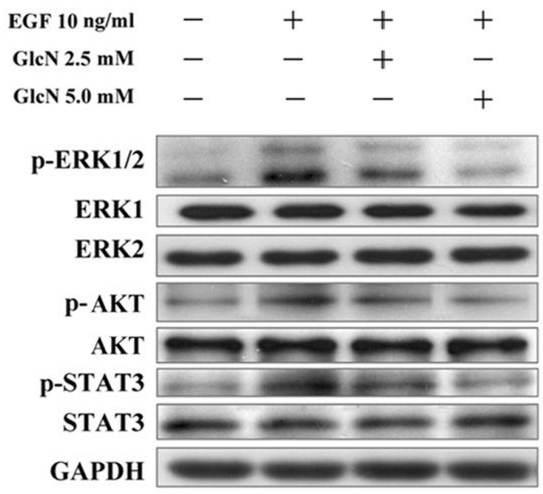
Effects of GlcN on EGF-stimulated phosphorylation of ERK, AKT, and STAT3. Human retinal pigment epithelial cell line (ARPE-19) cells were pretreated with 2.5 mM or 5.0 mM glucosamine (GlcN) under serum-free conditions for 24 h and incubated for a further 30 min in the presence or absence of 10 ng/ml epidermal growth factor (EGF). The same blot was probed with anti-phospho antibody or anti-total antibodies for ERK, AKT, and STAT3.

As shown in Figure 6, stimulation of the control cells by EGF resulted in enhanced phosphorylation of ERK1/2, AKT, and STAT3. Preincubation of the cells with GlcN in the medium diminished the EGF-dependent phosphorylation of ERK1/2, AKT, and STAT3 in a dose-dependent manner.

### GlcN suppressed EGF-induced increases in β-catenin

**Figure 7:**
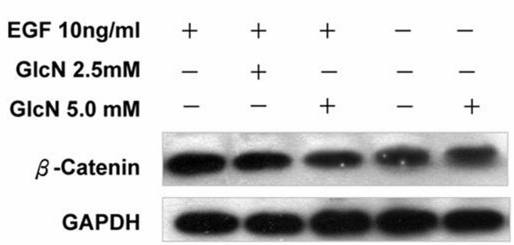
Glucosamine (GlcN) suppressed epidermal growth factor (EGF)-induced increases in β-catenin. The human retinal pigment epithelial cell line (ARPE-19) cells were cultured to 80% confluence, treated with 2.5 mM or 5 mM GlcN in serum-free medium for 24 h, then stimulated with EGF for further 24 h and probed with antibody against β-catenin.

We also performed western blot analysis to examine the effects of GlcN on the EGF-induced expression in β-catenin (Figure 7). GlcN had no effect on the expression of β-catenin in the absence of EGF, but it effectively suppressed the increased expression of β-catenin induced by EGF.

### Inhibition of EGFR phosphorylation in GlcN-treated RPE cells

**Figure 8:**
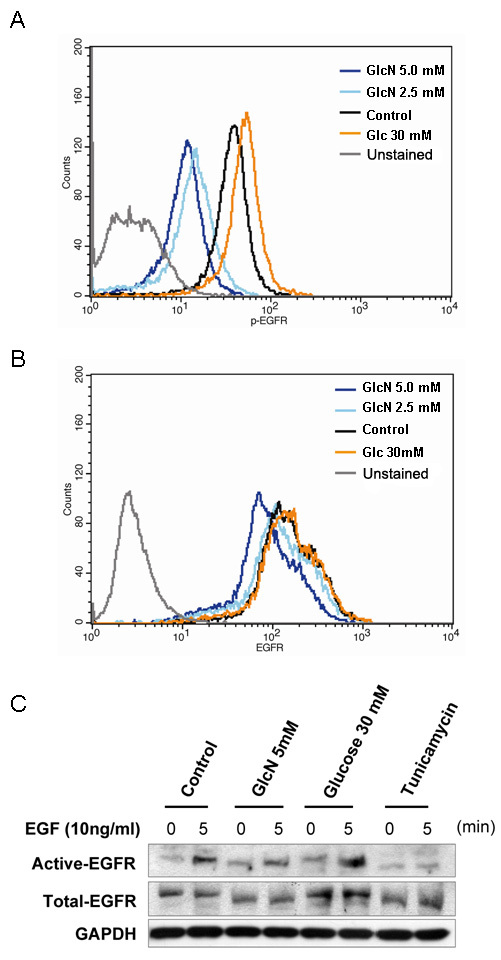
Effects of glucosamine (GlcN) on transactivation of epidermal growth factor receptor (EGFR). Human retinal pigment epithelial cell line (ARPE-19) cells were treated with 2.5 mM or 5.0 mM GlcN, or 30 mM glucose for 24 h, stimulated with 10 ng/ml EGF, and stained with PE-conjugated anti-total-EGFR antibody and Alexa-Fluor-647-conjugated anti-p-EGFR antibody (Y845). The levels of phosphorylated (**A**) and total EGFR (**B**) were analyzed by flow cytometry. The data are representative of at least three independent experiments. Cells cultured under serum-free conditions were used as the control. **C**: western blot analysis of ARPE-19 cells cultured to 80% confluence; treated with 2 μg/ml tunicamycin, 30 mM glucose, or 5 mM GlcN in serum-free medium for a further 24 h; then stimulated with EGF for 5 min.

Upon stimulation with EGF, the levels of phosphorylation of EGFR observed on the surface of cells treated with GlcN were lower than on the control cells (Figure 8A) and on cells that had been treated with 30 mM glucose as a positive control. There also appeared to be a slightly reduced level of total EGFR on the surface of the GlcN-treated cells (Figure 8B). Western blot analysis of the levels of total and phosphorylated EGFR (Figure 8C) showed a similar reduction in the total and phosphorylated form of EGFR on the surface after treatment with GlcN, whereas high levels of glucose increased the expression and phosphorylation of EGFR. Tunicamycin, on the other hand, had little effect on the total EGFR but markedly reduced its phosphorylation.

### Inhibition of N-Linked glycosylation in GlcN-treated RPE cells

**Figure 9:**
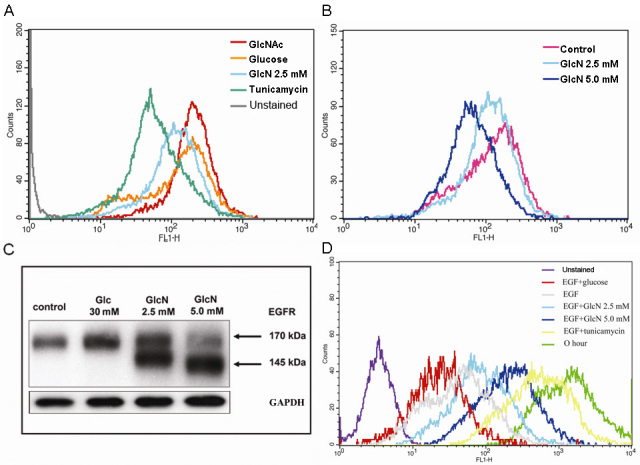
Effects of glucosamine (GlcN) on the expression of β-1,6-GlcNAc-branched N-glycan in the human retinal pigment epithelial cell line (ARPE-19) cells and the regulation of ARPE-19 cell proliferation by N-glycan branching. ARPE-19 cells at 80% confluence were treated with 2 μg/ml tunicamycin, 30 mM GlcNAc, 30 mM glucose, 2.5 mM GlcN, or 5 mM GlcN for 24 h. The cells were harvested, incubated with fluorescein isothiocyanate (FITC)–L-PHA (10 μg/ml), and L-PHA lectin binding was analyzed by flow cytometry. **A**: Binding of L-PHA lectin to GlcN-treated cells are compared with those in the positive (30 mM GlcNAc or 30 mM glucose) and negative (tunicamycin) control cells. **B**: The effect of GlcN on L-PHA lectin binding is dose-dependent. Cells were cultured in serum-free medium containing GlcN (2.5 mM or 5.0 mM) or high glucose (Glc, 30 mM) for 24 h. **C**: The expression of epidermal growth factor receptor (EGFR) in ARPE-19 cells treated with 2.5 mM or 5.0 mM GlcN or 30 mM glucose analyzed by western blot. D: CFSE staining of human donor retinal pigment epithelium (RPE) with various levels of N-glycan branching. The data are representative of at least three independent experiments.

Compared with the positive control cells, which had been treated with GlcNAc or glucose, cells cultured with GlcN for 24 h showed decreased binding of L-PHA (Figure 9A). A greater reduction in L-PHA binding was observed in the cells treated with 5.0 mM GlcN than in those treated with 2.5 mM GlcN (Figure 9B). Similarly, EGF receptors at 170 kDa (the molecular mass corresponding to the glycosylated form) were evident in the control cells as determined by western blotting; and treatment with 30 mM glucose increased the level of 170-kDa receptors after incubation for 24 h (Figure 9C). In contrast, when the medium was changed to one containing GlcN, a 145-kDa EGFR protein became increasingly apparent in a dose-dependent manner. Finally, we observed that human donor RPE cells with higher levels and greater branching of N-glycans on EGFR proliferated more rapidly than did cells with lower levels, as determined by staining with CFSE and flow cytometry (Figure 9D).

### Anti-proliferation effect of GlcN not from endoplasmic reticulum stress

**Figure 10:**
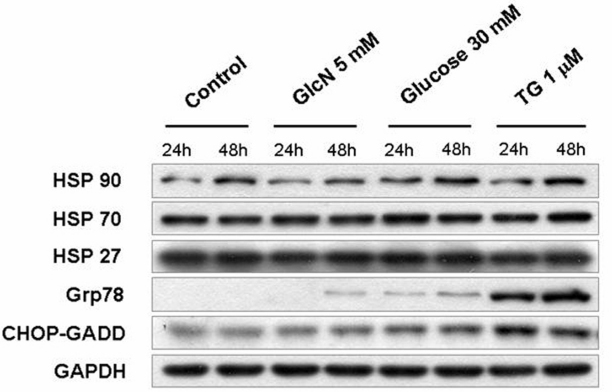
Effects of glucosamine (GlcN) on the expression of endoplasmic reticulum (ER) stress markers, Hsp27, Hsp70, Hsp90, Grp78, and CHOP-GADD. Human retinal pigment epithelial cell line (ARPE-19) cells were treated with 5 mM GlcN, 30 mM glucose or 1 μm thapsigargin (TG), a pharmacological ER stress inducers as a positive control, for 24 or 48 h. The expression of ER stress markers in ARPE-19 cells was analyzed by western blot.

To determine whether the effects of inhibition of N-Linked glycosylation of EGFR and anti-proliferation in GlcN-treated RPE cells were due to GlcN-induced nonspecific stress in the endoplasmic reticulum (ER), we evaluated the expression of Hsp90, Hsp70, Hsp27, Grp78, and CHOP-GADD (markers of stress in the ER) in GlcN-treated ARPE-19 cells. Western blot analyses showed only minor effects on the expression of Grp78, and CHOP-GADD when cells were treated with 5 mM GlcN, in contrast to the marked increases in their expression caused by treatment with thapsigargin (TG) or 30 mM Glucose (Figure 10). The expression of Hsp90 increased in the presence of 30 mM glucose but not GlcN. The expression of Hsp70 and Hsp27 was scarcely affected by the addition of any of the stimulants. These results indicated that at 5 mM, GlcN did not induce high levels of ER stress in the RPE cells, which suggested that the observed effects of GlcN on RPE cells were not nonspecific effects from ER stress.

## Discussion

The purpose of this study was to test the hypothesis that GlcN inhibits EGF-induced RPE proliferation, in vitro, and to explore whether the branching and levels of N-glycans on EGFR were involved in the mechanism. The results of the present study demonstrated for the first time that treatment with GlcN at concentrations of 2.5 mM and 5.0 mM significantly suppressed the EGF-induced proliferation of ARPE-19 cells in a dose-dependent manner, and slowed the cell-cycle progression of ARPE-19 cells through the G_1_–S and G_2_–M transitions without affecting cell viability. The antiproliferative effects of GlcN were confirmed in human donor cells. This study also provides evidence that GlcN modulated the level and branching of N-glycans on EGFR, suppressed EGFR transactivation, and reduced the phosphorylation of ERK, AKT, and STAT3. Finally, we demonstrated that GlcN mediated these effects through the EGF–EGFR signaling pathway.

### GlcN suppresses EGF-induced proliferation of ARPE-19 and human donor cells

Our findings with respect to the activation of RPE cell proliferation are consistent with those of previous studies, indicating that RPE cells are highly responsive to EGF [4]. Khaliq et al. [9] showed that EGF could stimulate the proliferation of RPE in vitro. Sugino et al. [8] demonstrated that EGF and its signaling pathways are critical factors that promote the survival, proliferation, adhesion, and migration of RPE cells in models of age-related macular degeneration. In addition, Chen et al. have shown that EGF promoted expression of integrin-alpha(5) and the subsequent proliferation and migration of ARPE-19 cells [12]. Our data also show that GlcN effectively suppressed the proliferation of ARPE-19 cells induced by EGF and added to the list of cells, the proliferation of which has been shown to be suppressed by GlcN [15].

### GlcN suppresses proliferation of ARPE-19 cells by delaying the cell cycle progression

The dose-dependent suppression of EGF-induced proliferation of ARPE-19 cells by GlcN observed in the present study could be partially explained by an effect on the cell cycle mediated by EGF–EGFR signaling. As we know, EGF is required for the G_1_–S transition and DNA replication in different cell types [16,17]. One study also found a novel EGF-sensitive checkpoint at which EGF-dependent cells undergo delay in the G_2_–M phase of the cell cycle before the activation of EGFR [18]. To determine whether a nonspecific response to stress in the ER contributed to the delay of cell growth, we examined the expression of Hsp90, Grp78, and CHO-GADD, both markers of ER stress. Our data showed that the expression of these markers was only minimally altered in response to GlcN, whereas their expression was markedly increased after exposure of the cells to high concentrations of glucose or thapsigargin. Although production of stress in the ER by GlcN cannot be absolutely ruled out by our data, these results suggest that the effects of GlcN did not produce high levels of nonspecific effects in the ER.

### GlcN delays expression of cyclins associated with progression of the cell cycle

To gain a better insight into the mechanism responsible for the delay of cell-cycle progression caused by GlcN, we evaluated its effects on several cyclins, CDKs, and CDK inhibitors. In mammalian cells, mitogens induce the expression of cyclin D and cyclin E, which are sequentially assembled and activated with CDKs, and reduce the amounts of the CDK inhibitor, p27, during G_1_. Cyclin D-associated CDKs and the cyclin E–Cdk2 complex then become active, phosphorylate, and inactivate Rb. After release from the repression of Rb, E2F and its target genes, such as those encoding cyclin A and several DNA replication enzymes, are expressed. These events mediate cell-cycle progression through G_1_ into S phase. Other studies have demonstrated the growth-inhibitory effects of EGFR inhibitors or anti-EGFR antibody [19]. We observed that GlcN caused the delayed expression of cyclin A and cyclin E and the delayed and prolonged expression of cyclin D, leading to the accumulation of cells in G_0_–G_1_ phase, delayed cell-cycle progression at the G_1_–S transition, and the reduced degradation of p27 in ARPE-19 cells. GlcN-treated cells also exhibited slowed progression through G_2_–M phase or delayed mitosis after release from an aphidicolin block. These results again suggest that GlcN affects EGF–EGFR signaling to inhibit the proliferation of EGF-dependent cells.

### GlcN reduces phosphorylation of EGFR and downstream signaling molecules

The effects of EGFR signaling on cell proliferation and survival are mediated by a complex network of intermediates, including mitogen-activated protein kinase, AKT, and STATs [20]. The binding of EGF to the extracellular domain of EGFR induces dimerization of the receptor and the activation of its intrinsic tyrosine kinase activity, leading to receptor autophosphorylation and the phosphorylation of tyrosine residues in various downstream signaling molecules, including ERK1/2, AKT, STAT3, and PKC [20]. Previous studies have shown that EGFR signaling mediated by the MEK/ERK and PI3K/AKT pathways is essential for RPE cell proliferation and survival [3]. One molecule downstream from EGFR, STAT3, can trigger the expression of target genes that are involved in cell-cycle regulation, including cyclin D, p21, p27, and c-Myc [21]. Here, we have demonstrated that GlcN slightly reduced the number of EGFR molecules on the surface of ARPE-19 cells and greatly reduced their phosphorylation at the Y845 residue. EGF is the most potent mitogen to cause the phosphorylation of Y845 [22]. Because EGF is the only mitogen we used to stimulate ARPE-19 cell proliferation, there should be no other nonEGFR ligands to influence the phosphorylation of the EGFR Y845 residue. Our results showing that the phosphorylation of the downstream molecules ERK, AKT, and STAT3 in EGF-stimulated ARPE-19 cells was also reduced, providing additional evidence that this EGFR pathway was involved. The results of our assessment of the inhibition of phosphorylation of STAT3 by GlcN are in good agreement with those of Chesnokov et al. in human prostate carcinoma cells, although GlcN induced apoptosis in that cell line, an effect that was not observed in the RPE cells in our study [15]. Our observation that GlcN can suppress the EGF-induced increase in expression of β-catenin further supports the conclusion that GlcN can effectively inhibit EGF–EGFR signaling.

### GlcN regulates EGF effects by modifying N-linked glycosylation on EGFR

N-linked oligosaccharides are crucial for the surface levels and functions of membrane-bound receptors for growth factors and cytokines [23]. The number and complex degree of the branching of N-glycans cooperate to regulate cell proliferation and differentiation [24]. Human EGFR contains 12 typical N-glycosylation consensus sites. N-glycan functions have also been extensively investigated, showing their involvement in receptor sorting, ligand binding, and dimerization [18]. Because GlcN is an inhibitor of the biosynthesis and processing of N-linked oligosaccharides, and because it causes dramatic and reversible changes in the nature of the lipid-linked oligosaccharides of glycoproteins [16], we hypothesized that reduction in the branching and levels of N-glycans on surface growth-factor receptors is a possible mechanism of action by which GlcN exerts its antiproliferative effect on ARPE-19 cells. Therefore, in the present study, we evaluated the N-glycosylation of EGFR by determining its binding of L-PHA, a plant lectin that specifically binds β-1,6-GlcNAc-branched N-glycans, and its molecular mass, which changes in a manner dependent on levels of N-glycosylation [21]. We have demonstrated for the first time that GlcN diminishes β-1,6-GlcNAc-branched N-glycan expression in a dose-dependent manner and modulates EGFR transactivation by reducing the level of N-glycans on EGFR.

### Possible mechanisms of action of GlcN

It has been known for some time that GlcN alters the protein and nucleotide contents of cells [25], disrupts the structure and function of the cellular membrane system [26], and modulates the levels of plasma-membrane gangliosides [27]. However, the mechanism for the antiproliferative effect of GlcN is incompletely understood; and the complexity of the pathways that regulate proliferation and the varying responses of different cell types to GlcN suggest that more than one pathway may be involved in its mechanism of action. Our study extends our knowledge about the mechanisms by which GlcN exerts its effects by demonstrating that, at least in these ARPE-19 cells, it modulates the levels and branching of N-glycans on the EGFR and inhibits EGFR transactivation and the downstream phosphorylation of STAT3, ERK, and AKT after stimulation with EGF.

### Limitations of the study

We confirmed the antiproliferative effects of GlcN in human donor cells; but we used ARPE-19 cells for the other experiments, such as the protein expression and lectin binding assays, because they are easier to culture than primary human cells. We should point out that although this cell line has been widely used for experiments on RPE cells, several studies have shown that the properties of ARPE-19 cells vary, depending on culture conditions [28,29]. This variability extends to the transcriptome of ARPE-19 cells [30], which is different from those of native adult, native fetal, and cultured fetal RPE cells, with 74 of 150 signature genes expressed at lower levels in ARPE-19 cells than in adult native RPE cells [31,32]. Therefore, our results should be considered provisional until they can be repeated with cultures of nontransformed human RPE cells. In addition, because the experiments described in this report have all been performed in vitro, further investigation is required to determine whether the results we have observed are confirmed in vivo.

### Conclusions

The results of our study may have bearing on the pathogenesis and treatment of PVR. Both previous findings and our own strongly suggest that EGF, which activates the EGFR signal transduction pathway, plays a key role in the pathogenesis of PVR. We have shown that GlcN can suppress the proliferation of RPE cells, the main target cells for the treatment of PVR. Our data support the hypothesis that GlcN effectively suppresses the EGF-induced proliferation of ARPE-19 cells through modulation of the branching and levels of N-glycans on surface proteins, including EGFR, and the inhibition of EGFR transactivation. The results of our in vitro investigation suggest the possibility that GlcN might play a role in the clinic as an agent for the treatment or prevention of RPE-mediated ocular proliferative disorders, such as PVR, and other EGF-dependent proliferative cell-growth disorders. Further research into this possible clinical application of GlcN is warranted.
